# Ultrasound criteria for risk stratification of thyroid nodules in the previously iodine deficient area of Austria - a single centre, retrospective analysis

**DOI:** 10.1186/s13044-018-0047-8

**Published:** 2018-05-09

**Authors:** Christina Tugendsam, Veronika Petz, Wolfgang Buchinger, Brigitta Schmoll-Hauer, Iris Pia Schenk, Karin Rudolph, Michael Krebs, Georg Zettinig

**Affiliations:** 1 Schilddruesenpraxis Josefstadt, Laudongasse 12/8, Vienna, AT-1080 Austria; 2 Division of Nuclear Medicine, Department of Biomedical Imaging and Image-guided Therapy, Medical University of Vienna, Vienna, Austria; 3 Schilddrueseninstitut Gleisdorf, Gleisdorf, Austria; 4 Department of Nuclear Medicine, Krankenanstalt Rudolfstiftung, Vienna, Austria; 5 Department of Nuclear Medicine, Sozialmedizinisches Zentrum Hietzing, Vienna, Austria; 6 Clinical Division of Endocrinology, Department of Medicine III, Medical University of Vienna, Vienna, Austria

**Keywords:** TIRADS, Sonography, Ultrasound, Nodule, Hypoechogenicity, Microcalcifications, Halo

## Abstract

**Background:**

We aimed to study the validity of six published ultrasound criteria for risk stratification of thyroid nodules in the former severely iodine deficient population of Austria.

**Methods:**

Retrospective, single centre, observer blinded study design. All patients with a history of thyroidectomy due to nodules seen in the centre between 2004 and 2014 with preoperative in-house sonography and documented postoperative histology were analyzed (*n* = 195). A board of five experienced thyroidologists evaluated the images of 45 papillary carcinomas, 8 follicular carcinomas, and 142 benign nodules regarding the following criteria: mild hypoechogenicity, marked hypoechogenicity, microlobulated or irregular margins, microcalcifications, taller than wide shape, missing thin halo.

**Results:**

All criteria but mild hypoechogenicity were significantly more frequent in thyroid cancer than in benign nodules. The number of positive criteria was significantly higher in cancer (2.79 ± 1.35) than in benign nodules (1.73 ± 1.18; *p* < 0.001). Thus, with a cut-off of two or more positive criteria, a sensitivity of 85% and a specificity of 45% were reached to predict malignancy in this sample of thyroid nodules. As expected, the findings were even more pronounced in papillary cancer only (2.98 ± 1.32 vs. 1.73 ± 1.18, *p* < 0.001). The six ultrasound criteria could not identify follicular cancer.

**Conclusion:**

Our findings support the recently published EU-TIRADS score. Apart from mild hypoechogenicity, the analyzed ultrasound criteria can be applied for risk stratification of thyroid nodules in the previously severely iodine deficient population of Austria.

## Background

Thyroid nodules are a common finding in any given population, with an estimated prevalence of 20-76% on ultrasound examination [1]. The vast majority of these nodules – regardless of whether they were initially palpable or incidental findings (e.g. upon carotid artery sonography) – are benign [2].

Therefore it is of high importance to discern truly benign thyroid nodules from those at higher risk [3]. Thyroid cancer is a tumor entity with steep increase in incidence, albeit with the majority of new cases belonging to the group of low risk tumor stages [4]. How to select the thyroid nodules for further assessment by fine needle aspiration is currently still a matter of debate [5]. Especially with the widespread use of high resolution neck ultrasound, many thyroid nodules are detected as incidentalomas [1, 2].

There is no typical sonographic pattern of thyroid cancer. Various sonographic criteria have been proposed to estimate the risk of malignancy in thyroid nodules. In 2009, two different proposals for a so-called TIRADS scoring system based on ultrasound nodule patterns were published [6, 7]. The concept was inspired by the widely used BIRADS system for assessing breast lesions, but partly been criticized for its complexity. It is still under debate whether this system can easily be applied to routine clinical use [5], and several modifications have been proposed during the last years [8–10].

In August 2017, the European Thyroid Association Guidelines for Ultrasound Malignancy Risk Stratification of Thyroid Nodules in Adults have been published [11]. Prior to this, the British Thyroid Association has published ultrasound features indicative of malignant nodule in its Guidelines for the management of thyroid cancer in 2014 [12], and the American Thyroid Association presented sonographic patterns suspicious for thyroid cancer in their 2015 guidelines [13], leading to some modifications of the Korean Thyroid Association guidelines in 2016 [14].

A number of studies evaluated the sensitivity and specificity of various criteria in patient samples from e.g. Korea [8, 15, 16], France [9, 10], or Poland [17]. We selected the following six criteria: mild hypoechogenicity [7–9, 11, 12], marked hypoechogenicity [7–9, 11, 12], microlobulated or irregular margins [7, 8, 9, 11, 12,], microcalcifications [7–9, 11, 12], taller than wide shape [7–9, 11, 12], and the absence of a thin halo [7, 12].

The presence of two or more of the six ultrasound criteria mild hypoechogenicity, marked hypoechogenicity, microlobulated or irregular margins, microcalcifications, taller than wide shape, and a solid component have been proposed to identify nodules at risk in a large Korean study evaluating more than 1600 patients including follow-up data which was the basis for the TIRADS Kwak score [8]. In the TIRADS French score system the four criteria irregular shape, irregular margins, microcalcifications, and marked hypoechogenicity are classified as highly suspect whereas mild hypoechogenicity in the absence of any of the four high suspicious features is the criterion for intermediate risk [11].

Comparable to France and several other European countries, Austria has been a moderately to even severely iodine depleted area with a high prevalence of endemic goiter, functional autonomies and cretinism, especially in the alp regions, for a long time [18]. In Austria, table salt has been iodized by federal law since 1963. The initial concentration of 10 mg KI per kg salt was increased to 20 mg/kg in 1990 when urine iodine secretion was still found to be in the mildly deficient range, currently it is 15-20 mg/kg. This strategy proved to be successful in greatly reducing the incidence of the above mentioned consequences of iodine deficiency [19].

Yet recent data indicate that iodine intake is still insufficient in at least part of the Austrian population, especially in pregnant women [20]. Due to decreased intake of table salt (as advocated for preventing hypertension) and the widespread use of not iodinated industrial salt, iodine intake might be insufficient even in the general population [20, 21].

The aim of our study was to assess six ultrasound criteria indicating thyroid cancer mainly published in the TIRADS Kwak score [8] and the TIRADS French score [9, 10] in the former iodine deficient Greater Vienna area. The study was conducted as a single centre, retrospective analysis of all nodules with postoperative histologic data available over the time course of 10 years (2004-2014). Five blinded experts rated the sonographic images according to the presence of published criteria. Diagnostic values of these criteria were then determined and compared to the published literature.

## Methods

### Setting and Study sample

The thyroid centre “Schilddruesenpraxis Josefstadt” is the largest private thyroid centre in Vienna and has been founded in 2004. We analysed all patients who were seen in this secondary care centre between October 2004 and December 2014 and identified all patients with the diagnosis of “history of thyroidectomy”. Among these 491 patients, 47 were operated due to Graves’ disease and excluded from analysis. There were 91 patients with thyroid cancer and 353 with benign thyroid nodules. Of the 444 patients seen postoperatively, 223 had preoperative thyroid sonography at the centre. From this sample another 28 patients were excluded due to the following reasons: poor image quality (12), very small microcarcinoma (14), original histological data not available (2).

Thus the initial study sample consisted of 195 patients: 45 papillary thyroid carcinomas (PTC), 8 follicular thyroid carcinomas (FTC), and 142 benign nodules (BN). Considering the medical history of each patient, the two unblinded investigators (GZ and VP) assigned all preoperative ultrasound images either to the thyroid cancer group or to the benign nodules group and anonymized all ultrasound images using the individual patient numbers given to all patients at the initial visit at the centre. GZ also selected the nodule that led to surgery in all patients with multinodular goitre. Images were available as electronic files since 2009, and before 2009 as prints.

### Retrospective expert review of the ultrasound images

Five experienced Austrian thyroidologists (CT, WB, BSH, KR, MK) met in April 2016 to review all preoperative sonographic patterns in a single session. They all have their focus on treating thyroid patients for many years, and their experience in thyroid sonography is up to 27 years.

The experts reviewed each nodule regarding the presence or absence of six ultrasound criteria given below. In a second step, they ranked the nodule as benign or malignant. Each expert wrote his or her assessment in an evaluation form (criterion 1-6 present or absent, nodule benign or malignant). Thereafter, the experts decided on the presence or absence of all six criteria and categorized the nodule as benign or malignant together. In case of disagreement between the experts, consensus was reached by discussion.

### Definition of ultrasound criteria of suspicion

The study was designed to evaluate the presence or absence of the following six criteria:

#### Mild hypoechogenicity

The nodule was classified as mildly hypoechogenic if the echogenicity was less than the thyroid parenchyma but more than the surrounding strap muscle.

#### Marked hypoechogenicity

The nodule was classified as marked hypoechogenic if the echogenicity was less than that of the surrounding strap muscle.

#### Microlobulated or irregular margins

The margin had many small lobules on the surface of a nodule or was infiltrative.

#### Microcalcifications

Defined as calcifications that were equal to or less than 1 mm in diameter and visualized as tiny punctate hyperechoic foci, either with or without acoustic shadows.

#### Taller than wide shape

The nodule was greater in its anteroposterior dimension than in its transverse dimension.

#### No thin halo

Absence of a thin hypoechoic rim around the nodule.

In partly cystic lesions, always the solid component was evaluated. Mild hypoechogenicity excluded marked hypoechogenicity and vice versa. Therefore, a maximum number of five criteria were possible in a single nodule.

### Statistics

Demographic data and nodule size are presented as mean ± standard deviation (SD). We compared them by chi-square statistics for categorical data and unpaired students t-test for continuous variables. The number of positive criteria was added for all nodules resulting in a minimum score of 0 and a maximum of 5 positive criteria (with mild hypoechogenicity and marked hypoechogenicity being mutually exclusive). Mean number of positive criteria in benign versus malignant lesions were compared by unpaired student’s t-test. A *p* < 0.05 was considered statistically significant. One-way ANOVA, followed by multiple t-tests with Bonferroni correction as post-hoc test (if appropriate), was used to compare mean numbers of positive criteria in the three subgroups of BN, PTC, and FTC.

The ability of the six ultrasound criteria to significantly discriminate between benign and malignant lesions was assessed by chi-square tests with Bonferroni correction for multiple testing. Thus, a *p*-value of < 0.0083 (0.05/6) was considered statistically significant.

Additionally the diagnostic values sensitivity, specificity, positive and negative predictive values (together with their respective 95% intervals) for each parameter and the expert opinion were calculated to predict the risk of cancer and PTC. The same diagnostic values were also calculated to compare the risk of malignancy in nodules with less than 2 vs. 2 or more criteria and less than 3 vs. 3 or more criteria, respectively. Statistical analysis was performed using SPSS Version 24 statistic software package.

## Results

### Demographic characteristics

Patients with cancer were significantly younger compared to the BN group (41 ± 11.9 vs. 49 ± 11.4 years; *p* < 0.001), and there was a lower rate of females among the cancer patients (79% vs. 89%, *p* = 0.063). An overview of the demographic characteristics of the subgroups (BN, PTC, FTC) is given in Table 1. PTC were significantly smaller than the nodules from the other groups.

**Table 1:** Detailed characteristics of the subgroups

	BN	PTC	FTC
Females	127/142 (89%)	36/45 (80%)	6/8 (75%)
Age (mean ± SD)	49 ± 11 years	42 ± 12 years	36 ± 11 years
Date of birth after 1 January 1963	63/142 (44%)	36/45 (80%)	6/8 (75%)
Maximum diameter of the nodule (mean ± SD)	28 ± 11 mm	20 ± 12 mm	31 ± 7 mm

The study sample included 103 patients (53%) born after 1963 (the year when iodization of table salt became mandatory in Austria). All but one patient were born before 1990 (when iodine content of table salt was increased from 10 to 20 mg/kg salt).

### Ultrasound criteria

Of the investigated malignancy suspicious criteria, all but mild hypoechogenicity were statistically different between benign and malignant lesions when compared by chi-square statistics. When comparing subgroups, the criterion no thin halo reached only borderline significance to predict PTC after Bonferroni correction for multiple comparisons (*p* = 0.01). Table 2 and 3 provide a detailed overview. Noteworthy, 23% of the carcinomas were isoechogenic.

**Table 2:** Chi-square statistics: Number (%) of positive criteria - BN versus cancer

BN vs. cancer:	BN	Cancer	Χ^2^	*p*-value
Mild hypoechogenicity	55/142 (39%)	19/53 (36%)	0.136	0.712
Marked hypoechogenicity	26/142 (18%)	22/53 (42%)	11.194	0.001
Microlobulated/ irregular margins	39/142 (27%)	29/53 (55%)	12.621	0.0004
Microcalcifications	10/142 (7%)	16/53 (30%)	17.894	0.00002
Taller than wide	17/142 (12%)	15/53 (28%)	7.503	0.006
No thin halo	99/142 (70%)	47/53 (89%)	7.375	0.007

**Table 3:** Chi-square statistics: Number (%) of positive criteria - BN versus PTC

BN vs.PTC:	BN	PTC	Χ^2^	*p*-value
Mild hypoechogenicity	55/142 (39%)	17/45 (38%)	0.013	0.909
Marked hypoechogenicity	26/142 (18%)	20/45 (44%)	12.583	0.0004
Microlobulated/ irregular margins	39/142 (27%)	27/45 (60%)	15.839	0.00007
Microcalcifications	10/142 (7%)	15/45 (33%)	20.394	0.00001
Taller than wide	17/142 (12%)	15/45 (33%)	10.993	0.001
No thin halo	99/142 (70%)	40/45 (89%)	6.582	0.010

The sensitivity, specificity, positive predictive value, and negative predictive value of each of the six criteria is given in Table 4 and 5 for both analyses (BN vs. cancer and the subgroup analysis). Sensitivity for the criterion no thin halo was 89%, all other criteria showed sensitivities of 60% and less. Several criteria showed a specificity of > 80%, whereas the most sensitive criterion no thin halo showed a specificity of only 30%.

**Table 4:** Diagnostic parameters for cancer

Cancer	Sensitivity	Specificity	PPV	NPV
Mild hypoechogenicity	36%	61%	26%	72%
Marked hypoechogenicity	42%	82%	46%	79%
Microlobulated/ irregular margins	55%	73%	43%	81%
Microcalcifications	30%	93%	62%	78%
Taller than wide	28%	88%	47%	77%
No thin halo	89%	30%	32%	88%

**Table 5:** Diagnostic parameters for PTC

PTC	Sensitivity	Specificity	PPV	NPV
Mild Hypoechogenicity	38%	61%	24%	76%
Marked hypoechogenicity	44%	82%	44%	82%
Microlobulated/ irregular margins	60%	73%	41%	85%
Microcalcifications	33%	93%	60%	82%
Taller than wide	33%	88%	47%	81%
No thin halo	89%	30%	29%	90%

### Mean number of positive criteria

Mean number of positive criteria were statistically different between BN and cancer (1,73 ± 1,18 versus 2,79 ± 1,35, respectively; *p* < 0.001, unpaired student’s t-test). There was also a significant difference between the three subgroups BN, PTC, and FTC (*p* < 0.001, one-way ANOVA). In the post-hoc tests (multiple t-tests with Bonferroni correction), PTC were statistically significantly different from BN (*p* < 0.001) as well as from FTC (*p* = 0.026), while mean number of criteria were comparable in BN and FTC. See Table 6 for details.

**Table 6:** Mean number of positive ultrasound criteria in BN and in all cancer patients as well as in the subgroups of PTC and FTC

	BN (*n* = 142)	Cancer (*n* = 53)	PTC (*n* = 45)	FTC (*n* = 8)
Mean number of criteria	1.73 ± 1.18	2.79 ± 1.35*	2.98 ± 1.32*	1.75 ± 1.04 **

* BN vs. Cancer: p < 0.001

* PTC vs. BN: p < 0.001, PTC vs. FTC: p = 0.026

** FTC vs. BN: *p* > 0.99, FTC vs. PTC: p = 0.026

### Calculated sums of positive criteria

Three out of 45 PTC (6.6%) did not show any of the criteria published in the literature. Of those, two were T1b and one was T1a. All three were labelled benign by the expert panel.

With a cut-off value of two or more positive criteria, 45 out of 53 malignant lesions (89%) were labelled correctly and 78 out of 142 benign lesions (55%) incorrectly as cancer. Thus, this cut-off resulted in a sensitivity of 89%[76-96], specificity of 45%[37-55], PPV of 34%[30-38], and NPV of 93%[85-97] to detect cancer based on ultrasound criteria when compared to benign lesions.

When increasing the threshold to three or more criteria, correct assignment of cancer to benign decreased while incorrect labelling of benign lesions as cancer dropped. See Tables 7, 8, 9, and 10 for details for both the PTC as well as the cancer group.

**Table 7:** Chi-square statistics of calculated sums of positive criteria – BN vs. cancer

BN vs. cancer	BN	Cancer	Χ^2^	p-value
≥1 criterion	120/142 (85%)	49/53 (93%)	2.10869	0.14646
≥2 criteria	78/142 (55%)	45/53 (85%)	14.89056	0.00011
≥3 criteria	36/142 (25%)	31/53 (59%)	18.79224	0.00001
≥4 criteria	10/142 (7%)	19/53 (36%)	25.29781	0.0000005
5 criteria	2/142 (1%)	4/53 (8%)	4.87687	0.02722

**Table 8:** Chi-square statistics of calculated sums of positive criteria – BN vs. PTC

BN vs. PTC	BN	PTC	Χ^2^	p-value
≥1 criterion	120/142 (85%)	42/45 (93%)	2.29850	0.12950
≥2 criteria	78/142 (55%)	40/45 (89%)	16.92502	0.00004
≥3 criteria	36/142 (25%)	29/45 (64%)	23.02780	0.000002
≥4 criteria	10/142 (7%)	19/45 (42%)	32.27589	0.00000001
5 criteria	2/142 (1%)	4/45 (9%)	6.15697	0.01309

**Table 9:** Sensitivity, specificity, PPV, and NPV of the number of criteria for cancer

Cancer	≥1 criterion	≥2 criteria	≥3 criteria	≥4 criteria	5 criteria
Sensitivity	93%	85%	59%	36%	8%
Specificity	16%	45%	75%	93%	99%
PPV	29%	37%	46%	66%	67%
NPV	85%	89%	83%	80%	74%

**Table 10:** Sensitivity, specificity, PPV, and NPV of the number of criteria for PTC

PTC	≥1 criterion	≥2 criteria	≥3 criteria	≥4 criteria	5 criteria
Sensitivity	93%	89%	64%	42%	9%
Specificity	16%	45%	75%	93%	99%
PPV	26%	34%	45%	66%	67%
NPV	88%	93%	87%	84%	77%

With this criterion, 16 nodules with a postoperative diagnosis of PTC were rated as benign. Those nodules were staged pT1a (7 nodules), pT1a(m) (2 nodules), pT1b (5 nodules), pT2 (1 nodule), pT3 (1 nodule).

### Pooled expert opinion

The experts diagnosed cancer with a specificity of 84%[76-90] and a sensitivity of 52%[38-65]. PPV was 58%[47-69] and NPV 80%[75-84]. The diagnostic values of the pooled expert opinion for PTC were: sensitivity 64%[49-78], specificity 78%[70-85], PPV 48%[39-58], NPV 87%[82-91]. Of the 16 PTC falsely labelled as benign by the experts staging was pT1a in 7, pT1b in 5, pT1b(m) in 1, and pT3 in 1 lesion. 6 fulfilled 3 or more positive ultrasound criteria. On the other hand experts rated 7 lesions fulfilling only 2 ultrasound criteria correctly as carcinomas. 8 lesions were missed by both methods (5 fulfilling 1 and 3 fulfilling 2 ultrasound criteria).

## Discussion

In light of the widespread use of ultrasonography and frequent findings of thyroid incidentalomas, strategies that systematically and reliably identify those thyroid nodules with a higher risk of malignancy are highly warranted.

In iodine deficient areas like Austria the spectrum of thyroid malignancies differs from iodine sufficient areas with a relatively higher proportion of follicular carcinomas and the occurrence of anaplastic carcinomas in multinodular goiters (which almost disappeared after the introduction of iodization of table salt) [22].

We therefore conducted a retrospective analysis of sonographic images from thyroid nodules with available postoperative histological data from Austrian patients (mainly from Vienna and surroundings). Since the published sonographic criteria relate to papillary carcinomas only, we also calculated a statistical analysis restricted to PTC vs. benign lesions.

At the time the study was designed, several criteria were repeatedly studied and published [7–9, 12]. According to previously published reports, we analyzed mild hypoechogenicity, marked hypoechogenicity, microlobulated or irregular margins, microcalcifications, and a taller than wide shape. As retrospective assessment of the composition of a nodule (solid, mainly solid, mainly cystic) in two dimensional images seemed problematic to us, we decided not to include this criterion in our analysis. These five criteria are also among the proposals for the TIRADS French [9] and the recently published EU-TIRADS scoring system [11] and are included in the system proposed in the recently published ATA 2015 guidelines [13]. Due to the rather small sample size, however, we decided to restrict our analysis to applying the most widely used criteria without applying a formal scoring system. In addition, we evaluated the absence of a thin perinodular halo. There is no hard evidence from studies, but the halo sign traditionally has been regarded as an important ultrasound sign for the risk stratification of thyroid nodules in central Europe [23, 24] and is still discussed as a suggested feature for thyroid cancer in several diagnostic algorithms [12, 25, 26]. Noteworthy, the halo sign has also recently been discussed also in the European Thyroid Association Guidelines for Ultrasound Malignancy Risk Stratification of Thyroid Nodules in Adults [11] and is one of the nine sonographic features initially identified in the white paper of the ACR TIRADS Committee [26]. Our findings indicate that the absence of a thin halo is also helpful for the diagnosis of thyroid cancer, at least in the former severely iodine depleted population of Austria.

Overall we found a good accordance of the published risk markers with the lesions evaluated in this sample. Our findings support the validity of the TIRADS Kwak criteria [8], the ATA 2015 criteria [13] as well as the TIRADS French [10] and the EU-TIRADS [11] criteria for the former severely iodine deficient area of Austria. One notable exception is the criterion of mild hypoechogenicity. In contrast to marked hypoechogenicity, mild hypoechogenicity could not discriminate between benign and malignant lesions in our sample. This is different to TIRADS Kwak [8]. ATA 2015 does not discriminate between mild and marked hypoechogenicity. In EU-TIRADS, mild hypoechogenicity indicates intermediate risk but no high risk for thyroid cancer [11], indicating that also in the previously iodine deficient population of France, mild hypoechogenicity is not that strict criterion for malignancy as in iodine sufficient regions such as Korea.

There are several possible reasons for this finding. Firstly, all the nodules included in this sample were considered to be reason enough to operate on the patient. Reasons leading to the operation decision were not systematically recorded, but included suspicious results in fine needle aspiration, nodule size, local complaints, patient’s wish, and also suspicion of malignancy in ultrasound. Thus, unambiguously benign lesions were underrepresented or (e.g. simple cysts) even absent from the study sample.Yet another possible reason for this finding is the effect of iodine deficiency that might results in changes in thyroid tissue that are only partially reversible upon iodine supplementation. In a large French sample (France being a formerly iodine deficient region), mild hypoechogenicity conferred only intermediate risk for thyroid cancer [27], in contrast to the iodine sufficient region of Korea.

Of note, 48% of the patients included in the study were born before the year 1963, when iodization of table salt was introduced in Austria and therefore spent at least parts of their early lives in severe iodine deficiency. Only one of the patients was born after 1990 when iodization of table salt in Austria was doubled to 20 mg/kg, because iodine supplementation was still considered insufficient [27]. The different iodine status in Austria and France could also be the explanation mild hypoechogenicity being classified as an intermediate risk factor in the TIRADS French study, but not discriminating in our sample.

A third European country with former iodine deficiency is Poland [28]. Recently an evaluation of four TIRADS classification systems in Polish multinodular goiter patients was published suggesting TIRADS Kwak as a suitable and practicable tool for this patient group. [17].

There is no single ultrasound feature which could reliably distinguish benign from malignant lesions. Some markers (e.g. microcalcifications) have a high specificity but insufficient sensitivity and vice versa. Thus, the combination of several features enhances the diagnostic value of sonography. In a recent Korean study [15], performed on a very large sample of thyroid nodules 10-19 mm in size, a head to head comparison of six risk stratification systems proposed in the literature was performed and yielded, as would be expected, different diagnostic values. Application of TIRADS French (using the number of positive criteria with a cut-off of two or more criteria being present for proposing fine needle aspiration) resulted in a sensitivity of 95% and a specificity of 52%, respectively. In the sample presented here, the cut-off value of two or more positive criteria for PTC resulted in a roughly comparable diagnostic performance (with slightly lower sensitivity of 89% and specificity of 45%). Other stratification systems resulted in even higher sensitivity at the cost of lower specificity.

On the other hand, the pooled expert estimation of malignancy risk yielded sensitivity and specificity very similar to the 3 criteria cut-off condition. Thus, 35% of PTC were mislabelled as benign from the expert panel (although the PTC misjudged as benign were all but one postoperatively staged as pT1).

The experts diagnosed cancer with a specificity of 84%, but the sensitivity was only 52%. For the diagnosis of PTC, the expert’s sensitivity of 64% was only slightly higher. These findings indicate that in real life setting, the accurate differential diagnosis of nodules still remains difficult. Our five thyroidologists, who have long-time experience in interpreting thyroid ultrasound, did not predict malignancy accurately. Using a number of at least two positive ultrasound criteria to define the risk of malignancy yields a higher sensitivity but a lower specificity than expert judgement.

Our findings suggest that mild hypoechogenicity should be clearly differentiated from marked hypoechogenicity. The role of mild hypoechogenicity as a malignancy marker has to be clarified in relation to iodine status: In the sample studied here, in opposition to marked hypoechogenicity - which was significantly more frequent in PTC - mild hypoechogenicity didn’t even show a trend towards higher frequency in PTC. On the other hand, the absence of a thin halo added diagnostic value in the sample presented here and might be worthy of consideration when evaluating nodules for possible malignancy.

The 2015 American Thyroid Association Management Guidelines for Adult Patients with Thyroid Nodules and Differentiated Thyroid Cancer [13] have been published after a long debate including a revised concept for ultrasonographic risk stratification of thyroid nodules. These guidelines do not distinguish between mild and marked hypoechogenicity. Hypoechogenic solid nodules without other risk patterns are classified as intermediately suspicious with a malignancy risk of 10-20%. In our study sample, however, the criterion hypoechogenicity was not found more often in cancer than in benign lesions.

The six criteria were not helpful for diagnosing follicular cancer. As presented in Table 6, the mean number of ultrasound criteria of FTC did not differ from BN, but from PTC. In these patients, the “nodule in nodule sign” could be a criterion for future studies [29]. If, as sometimes is suggested, the scintigraphic pattern (with reduced versus isointense activity) would be helpful in risk stratification of those nodules, is currently unclear. The sample did not include any patient with medullary thyroid cancer. Most probably this is because in Vienna nearly all patients with medullary thyroid cancer are followed up in a single tertiary centre after surgery.

Strengths of the study include the assessment by five thyroid experts in a systematic way and the availability of histological data of all the included nodules. Limitations of the study include the retrospective study design, that only B-mode images were used, the lack of information on the actual iodine status of the patients, the rather small sample size and the single center study design.

## Conclusions

In conclusion, we report validity of published ultrasound malignancy risk markers of thyroid nodules in the formerly severely iodine deficient area of Austria for the first time. Our findings support the EU-TIRADS scoring system. With the exception of hypoechogenicity, ultrasound criteria as described in the literature were applicable with good sensitivity for risk adjustment of thyroid nodules in this secondary care setting. Additionally, the missing halo sign was a sensitive malignancy marker in this sample, which might be useful in a screening setting.

## Abbreviations

BN: Benign nodules
FTC: Follicular thyroid cancer
PTC: Papillyry thyroid cancer
SD: Standard deviation
